# Identification of heavy work investment antecedents: a research on digital leadership

**DOI:** 10.3389/fpsyg.2025.1588412

**Published:** 2025-05-07

**Authors:** Burcu Turan-Torun, Onur Oktaysoy, Mehmet Selman Kobanoglu, Ethem Topcuoglu, Muhammed Akif Yenikaya, Vurgun Topcuoglu, Selen Uygungil-Erdogan

**Affiliations:** ^1^Faculty of Tourism, Van Yuzuncu Yil University, Van, Türkiye; ^2^Faculty of Economics and Administrative Sciences, Kafkas University, Kars, Türkiye; ^3^Faculty of Political Sciences, Samsun University, Samsun, Türkiye; ^4^Academy of Civil Aviation, Giresun University, Giresun, Türkiye; ^5^Faculty of Economy, Administrative and Social Sciences, Istanbul Nisantasi University, Istanbul, Türkiye; ^6^Kadirli Faculty of Applied Sciences, Osmaniye Korkut Ata University, Osmaniye, Türkiye

**Keywords:** digital leadership, heavy work investment, job performance, job satisfaction, career satisfaction

## Abstract

**Introduction:**

In today’s world, which is referred to as the era of digital transformation, the expectations from the leader role are changing significantly and digital leadership understanding draws attention as a reflection of these expectations. This study examines the impact of digital leadership on employees’ job performance, job satisfaction and career satisfaction and examines the mediating role of Heavy Work Investment (HWI) in this relationship. Within the framework of the sub-dimensions of HWI such as Time Commitment (TC) and Work Intensity (WI), the study investigates how employees’ investment in their work is shaped and the guiding role of digital leaders in this process.

**Methods:**

This study, which aims to determine the mediating role of HWI in the effect of digital leadership on job performance, job satisfaction and career satisfaction of employees, was conducted with 393 employees working in SMEs operating in the IT sector. The data obtained by convenience sampling methods were analyzed with Smart-PLS program. The study was shaped on the axis of structural equation modeling.

**Results:**

The findings of the analysis reveal that digital leadership has a significant effect on employees’ job performance, job satisfaction and career satisfaction. In addition, it has been determined that HWI creates different mediation mechanisms in terms of its sub-dimensions TC and WI in the relationship between digital leadership and employees’ job performance, job satisfaction and career satisfaction.

**Discussion:**

The research findings reveal that digital leadership has positive effects on employees’ job performance, job satisfaction and career satisfaction and that HWI plays a partial mediating role in this process. It is noteworthy that while WI is found to be a strengthening factor in this relationship, the effect of TC on job satisfaction and career satisfaction is not significant. This suggests that TC may lead to negative consequences such as burnout and stress instead of increasing employees’ motivation and performance. Moreover, practices that increase employee engagement in the digital transformation process appear to play a critical role in maintaining organizational efficiency and employee well-being in the long run. While the findings are in line with the existing literature, they suggest that a deeper understanding of the interaction dynamics between digital leadership and HWI is needed.

## 1 Introduction

Digital transformation has become one of the key elements of gaining competitive advantage in today’s business world. Technological advances radically change the management approaches, organizational processes and employee expectations of businesses. In this context, the concept of digital leadership gains importance as a strategy that not only manages technological transformation processes, but also facilitates employees’ adaptation to change, increases motivation and increases productivity (Sagbas et al., 2023). Digital leaders encourage employees to think innovatively, while optimizing business processes and supporting performance improvement. Research shows that effective digital leadership practices significantly increase employee engagement and job performance (Chatterjee et al., 2023).

HWI stands out as a critical variable that expresses the amount of time and energy employees spend on work processes. HWI is divided into two main components, situational and external, and is a determinant of employees’ reaction to work and long-term job performance (Tziner et al., 2019). Among the sub-dimensions of HWI, workaholism increases the risk of burnout syndrome, while work engagement is associated with higher job satisfaction (Snir and Harpaz, 2021). At this point, the question of how HWI mediates the relationship between digital leadership and employees’ job satisfaction, job performance, and career satisfaction gains importance.

Unlike traditional leadership approaches, digital leadership is defined as a leadership approach that encourages technology-driven innovation, guides employees to adapt to digital transformation, and increases organizational efficiency by using digital tools effectively (Benitez et al., 2022). Although the impact of digital leaders on employees’ job performance, job satisfaction and career satisfaction has received increasing academic attention in recent years, there are significant gaps in the literature on the mechanisms through which this relationship is shaped (Topcuoglu et al., 2023b). In particular, how the concept of HWI, which is defined as the intensive investment of employees in work, plays a mediating role in the relationship between digital leadership and employees’ work outcomes is a topic that has not yet been sufficiently researched. This study will determine the impact of digital leadership practices on employees’ job performance, job satisfaction and career satisfaction and how HWI plays a mediating role in this process (Houlfort et al., 2014). It will also provide strategic guidance on how leaders should balance their employees’ work investments in the digital transformation process. In developing digital leadership practices, it will help businesses understand whether employee investment in their work is a motivating factor or a burden that leads to burnout. At the same time, it will be an important practical contribution, emphasizing the need for digital leaders to develop strategies to protect the well-being of employees while encouraging them to invest more in their work.

In this context, the study aims to make a unique and valuable contribution to the relevant literature in both theoretical and practical terms. Academically, it brings a new perspective to the literature by addressing the concepts of digital leadership and HWI together, while in terms of practice, it aims to guide managers on how digital leadership practices should be shaped in the era of digital transformation. In this context, SMEs operating in the information technologies sector in Turkiye/Adana province were selected as the sample. A total of 393 employees were reached by convenience sampling method. The data obtained were analyzed by structural equation modeling through Smart-PLS program. In the light of the findings, the mediating role of heavy work investment in the effect of digital leadership on employees’ job performance, job satisfaction and career satisfaction was determined as low and partial.

## 2 Conceptual framework

### 2.1 Digital leadership

Developments in the business world enable leaders to manage more comprehensive and complex processes with various technologies and offer significant advantages in managing much more challenging processes than traditional methods (Benitez et al., 2022). However, these technologies alone cannot be sufficient in terms of organizational advantage, and new skills and qualities are demanded from the leader in terms of utilizing these technologies for a sustainable organizational structure (Zeike et al., 2019). The digital leadership approach, which emerged in response to this demand, stands out as an understanding that defines the leaders of the digital age (Erhan et al., 2022). Leaders in the digital age should be able to follow digital transformation, make strategic decisions based on data analysis, comprehend technological trends, transform their organizations in line with the requirements of the change and create a flexible organizational structure, in short, lead digital transformation (Demir et al., 2025).

Some of the definitions of the concept of digital leadership are as follows; a digital leader is “a leader who can direct the organizational structure, access information through digital channels, lead digital transformation, foresee changes that will form the basis for the organizational goals to be achieved and establish relationships” (Sheninger, 2019). In a different definition, digital leadership is defined as “a leadership style that can fulfill the roles attributed to the leader in the digital age by making use of digital tools, mastering digital technologies, and managing the organization” (Borah et al., 2022). Zhu et al. (2022) define digital leadership as a combination of five different leadership approaches: thought leadership, creative leadership, visionary leadership, curious leadership and wise leadership.

Digital leadership, which reflects the leadership understanding of our age shaped by technological developments, includes the leader’s ability to operate effectively in the digital world (AlAjmi, 2022). While coordinating members’ online activities, digital leaders can also effectively manage organizational strategies, problem-solving methods, and decisions made in the digital arena (Weber et al., 2020). The main feature that distinguishes the digital leader approach from classical leader approaches is not their skills, expertise, professionalism or technological superiority, but the fact that they know what standard and how technology should be used in managing business and organization (Karafakioglu and Afacan Findikli, 2024). What should be underlined here is that in digital leadership, it is not the leader’s competence in digital technologies that is important, but the capacity to use them in line with organizational interests. This is a critical capability attributed to the leader for businesses to succeed in the digital age (Topcuoglu et al., 2023b).

Digital leadership is the art of understanding how technology-oriented advances brought by the age can be adapted to organizational success and performance, taking the necessary steps for this, and convincing members of this process (Topcuoglu et al., 2023a). This leadership approach emphasizes the ability to use technology as a strategic tool, to increase the competitive capacity of the organization with innovative solutions, and to ensure that members as well as themselves can benefit from digital technologies and platforms in a qualified way (Klein, 2020).

A multidisciplinary understanding and integration is necessary for organizations to successfully carry out the digital transformation process, which is still taking shape (Erhan et al., 2022). Digital leaders play a very important role in realizing this integration. In particular, in order to adapt to the rapid change in the axis of digitalization, the transition to this leadership approach is becoming a necessity for organizations (Benitez et al., 2022).

Digital leaders, who should focus on the organizational transformation and development process, should have the knowledge, equipment, determination and competence to deal with all kinds of problems, obstacles, resistance and incompatibility that may be experienced in this process (Büyükbeşe et al., 2022). With the new methods, plans and programs required for the transition process in question, the adaptation of the existing system to the system to be created should be ensured, and the members of the organization should be made ready throughout this whole process. These requirements require the digital leader to be persuasive, visionary, solution-oriented, sharing and transparent, open to innovations, fast learner and adaptable (Abbu et al., 2022). These roles expected from digital leaders are often confused with the leadership approach referred to as “transformational leadership” in the literature. However, digital leadership refers to the combination of transformational leadership approach and digital technology competence, and therefore it can be said that it is a more comprehensive form of transformational leadership (Benitez et al., 2022).

It is worth noting that literature on digital leadership is generally positive. Although this positive situation is often emphasized in the current study, digital leadership also has negative effects such as unrealistic expectations, lack of personal interaction, technostress or digital fatigue (Alkhayyal and Bajaba, 2024; Ertiö et al., 2024; Rademaker et al., 2025). At the same time, excessive digital monitoring increases employees’ fear of making mistakes and creates tension in the work environment, leading to burnout (Li et al., 2025). In this respect, the importance of balancing the work demands with the work resources created by the leaders once again emerges.

### 2.2 Heavy work investment

Snir and Harpaz (2012) conceptualized employees’ high time and intensive work investment in work as HWI. HWI is a scientifically based construct that includes both workaholism and work engagement (Loscalzo et al., 2023). In this direction, it is seen that two models of HWI have emerged over time.

According to the model proposed by Schaufeli (2016), the concept of HWI has two basic dimensions. Workaholism depending on time and work engagement depending on intensive work represents the two dimensions of the HWI concept (Loscalzo et al., 2023). In this model, workaholism refers to a “bad” and work engagement refers to a “good” kind of HWI (Schaufeli, 2016). Workaholism is the obsessive overworking of employees. In addition, workaholics have various negative behaviors associated with neurotic personality traits (Van Beek et al., 2014). Work engagement refers to the well-being of the HWI. Work engagement is defined as a positive, rewarding psychological state characterized by dedication, commitment, and physical and intellectual energy that creates a healthy well-being environment for employees (Tecau et al., 2020). This model is supported by the Job Demands-Resources (JD-R) Theory (Demerouti et al., 2001), which proposes the use of workaholism and work engagement scales to measure HWI (Schaufeli, 2016). Job demands initiate a process of deterioration in an employee’s health due to prolonged experience of stress and emotional exhaustion, while work resources create a motivational process where employees want to be more engaged in their work (Rattrie et al., 2022). The HWI framework developed here focuses on both health impairments and motivational processes identified in the JD-R model and considers HWI to be a continuum (Tabak et al., 2021).

In the second model created by Snir and Harpaz (2012), it is seen that six different subtypes of HWIs, two situational and four external, are motivated and formed. Workaholism and work engagement subtypes are under the heading of external antecedents; in short, there is a tendency arising from individual characteristics. Situational antecedents arise from external or environmental situations that cannot be controlled by the individual. Later on, the number of subtypes was reduced from six to four in line with the studies and evidence obtained (Snir et al., 2023). Workaholics constitute the first subtype of external antecedents. Conceptualized by Oates (1971), workaholism was identified with alcohol addiction and defined as a deviant type of work addiction (Houlfort et al., 2014).

Workaholism is used for people who put their desire to work above their health, personal happiness, social relationships or social life. At the same time, the concept refers to people who tend to work excessively to the extent that they pose a danger to themselves (Snir and Harpaz, 2009). The second subtype of external antecedents consists of employees who are dedicated to work. Work engagement is defined as a positive, satisfying, work-related state of mind characterized by vitality, dedication and concentration (Bakker and Demerouti, 2017). It means that the employee is strongly involved in his/her work and experiences a sense of significance, enthusiasm and challenge (Schaufeli, 2016). Even if people feel tired after a long day at work, they perceive it as a pleasant experience that comes with a sense of accomplishment (Snir et al., 2023).

The first subtype of situational antecedents consists of employees who are overworked due to organizational structure or job characteristics. This group includes situations where management implicitly or explicitly forces employees to work long hours (Snir and Harpaz, 2009). In particular, the structure of technology companies that always require interaction and professions such as police, military service, and medicine are included in this category (Tziner et al., 2019). There is a structure in which those who do not act in accordance with the situation are isolated within the organization. The second subtype of situational antecedents is the needy. People are expected to meet their financial needs by working more to meet their own or their relatives’ needs (Afota et al., 2025). Working long hours at a single workplace or trying to earn more income by working more than one job also points to this situation (Kaygin et al., 2023). The characteristics of the two antecedents and four subtypes of HWI are briefly presented in Table 1.

**Table 1:** Four subtypes of heavy work investors.

Situational heavy work investors	External heavy work investors
1. Those affected by organizational culture, high-tech workers, hospital doctors, etc.	1. Workaholics–those who are addicted to their work.
2. Those who are in need must support a large family, pay off debts, etc.	2. Those who are dedicated–those who have a great passion for their work.

Source: Snir et al. (2023).

The second model created by Snir and Harpaz (2021) is supported by attribution theory (Weiner, 1985). In later studies, it is seen that the model created by Snir and Harpaz (2012) was developed especially with JD-R Theory (Tziner et al., 2019). When HWI is examined, it is seen that there are more than one subtype depending on the external characteristics of the person, organizational structure and need. While the past experiences and tendencies of the person direct the person to HWI, on the other hand, organizations exhibit facilitating actions that unintentionally encourage HWI of their employees (Astakhova and Hogue, 2014). Considering the increasing competition in today’s business world, it is thought that the tendency toward HWI will increase. In this context, it is generally stated that intensive work investors with high enjoyment show the best psychological and health outcomes, while those based on financial needs show the worst outcomes. The other two types, overworked and workaholics, are observed to be positioned in between (Tziner et al., 2019). Snir and Harpaz (2012) propose the scale developed by Brown and Leigh (1996) for the measurement of HWI. According to Brown and Leigh (1996), Time Commitment (TC) is recommended for those who devote more time to the work of the organization, while Work Intensity (WI) gains importance for employees who adopt intensive work during working time.

In this study, the model proposed by Snir and Harpaz (2012) was followed to analyze digital leadership and organizational variables as potential antecedents and consequences of HWI. This choice was made with the inclusion of leadership in the study and the possibility of showing more explanations for organizational orientation in the model.

### 2.3 Job performance

Job performance is a critical concept for organizations that refers to the level of efficiency, effectiveness and success of employees at work (Rizaie et al., 2023). Job performance, which is defined in the literature as the degree to which employees fulfill their job roles within a certain period of time, is generally considered in three dimensions: task performance, contextual performance and adaptive performance (Robbins et al., 2010). While task performance covers the activities performed by the employee in accordance with the job description, contextual performance includes the employee’s contribution to teamwork, organizational citizenship behaviors and the social environment at the workplace. Adaptive performance includes the employee’s ability to adapt to changing work conditions and problem-solving skills (Diamantidis and Chatzoglou, 2018). Job performance is influenced by employees’ knowledge, skills and motivation, as well as external factors such as work environment, leadership style and organizational support, and plays an important role in determining both individual and organizational success (Gong et al., 2019).

The theoretical background of job performance has been shaped within the framework of organizational behavior, management science and psychology disciplines. Among the most important theoretical models explaining this concept are Goal Setting Theory (Latham and Locke, 1979) and Expectancy Theory (Vroom, 1964). Goal-setting theory argues that employees exhibit higher performance when they have clearly defined, low complexity and optimally challenging goals, as well as when they receive effective feedback from the leader (Celik et al., 2023), while Expectancy theory suggests that individuals tend to perform to the extent that they believe that they will achieve what they hope to achieve (Fu and Deshpande, 2014). Job Characteristics Model (Hackman and Oldham, 1980) states that the presence of meaning, autonomy and feedback elements in the employee’s job will increase performance.

When the effects of high job performance on organizations are examined, it is seen that it is directly related to increased productivity, customer satisfaction, innovation capacity and competitive advantage (Yao et al., 2022). Employees with high performance contribute more to the achievement of organizational goals and increase efficiency in the overall functioning of the organization. In addition, high job performance is considered a critical element for sustainable growth and financial success in organizations and is seen as an important component of the development of workplace culture (Abolade, 2020). From an individual perspective, it is frequently emphasized in the literature that job performance is a determining factor on factors such as career development, salary increases, job satisfaction and psychological well-being. High-performing employees have more access to professional development and promotion opportunities, while low-performing individuals may face problems such as job insecurity, loss of motivation and stress (Wang et al., 2021). Moreover, low job performance increases employees’ risk of burnout syndrome and reduces their organizational commitment in the long run (Mokhtar et al., 2021). Current research reveals that job performance is shaped not only by individual competencies but also by factors such as organizational support, leadership style, and work-life balance (Somu et al., 2020).

### 2.4 Job satisfaction

Job satisfaction is defined as a multidimensional concept that expresses the degree of psychological and emotional satisfaction that employees feel with their jobs and is affected by many factors at the individual and organizational levels (Liu et al., 2022). This concept is directly related to elements such as job content, working conditions, leadership practices, compensation, career development, job security and organizational support (Sakarji et al., 2021). Job satisfaction is an important factor that shapes employees’ attitudes toward work and determines their motivation, commitment and overall job performance. High job satisfaction increases employees’ morale and motivation, while significantly reducing intention to leave and absenteeism rates (Loo et al., 2024). From the perspective of organizations, it is observed that employees with high job satisfaction engage in more productive activities and contribute to the positive development of the work environment by exhibiting organizational citizenship behaviors (Sypniewska, 2014).

When the literature is examined, it is seen that the factors affecting job satisfaction are generally addressed at individual and organizational levels. Individual factors include personality traits, demographic variables, and employees’ career expectations. Studies reveal that individuals with a positive emotional structure report higher job satisfaction, while neurotic individuals have lower job satisfaction (Koustelios, 2001). Organizational factors consist of elements such as wage policies, working conditions, leadership style, career opportunities, and organizational support. The work environment and leadership style play a critical role in job satisfaction, and the level of job satisfaction increases in organizations where employees feel valued, participate, and receive support (Ong et al., 2020). In addition, the availability of career development and promotion opportunities is seen as one of the most important factors that increase employees’ job satisfaction (Rukh et al., 2015). On the other hand, one of the most important studies conducted to determine the factors affecting job satisfaction in a theoretical sense is Herzberg’s Dual Factor Theory. According to this theory, the factors that shape job satisfaction in employees are divided into two as motivation and hygiene factors. While motivation factors include elements such as success, recognition and development opportunities, hygiene factors such as wages, working conditions and job security come to the fore (Gazi et al., 2024).

The effects of high or low levels of job satisfaction are not limited to the process between the individual and the organization but can also have important consequences on individuals’ lives outside of work in the long term (Rahman et al., 2017). At this point, various studies have shown that individuals with high job dissatisfaction experience emotional depression, headaches, sleep problems, and neurological problems, and that their physical and mental health, stress levels, quality of life, and social relationships outside of work are significantly negatively affected in the long term (Schaufeli et al., 2009).

### 2.5 Career satisfaction

Career is a process shaped by the professional experiences, skills and achievements of an individual throughout his/her life and includes not only the individual’s progress in business life but also factors such as professional identity development, learning process and job satisfaction (Arthur et al., 2005). While traditional career approaches focus on elements such as hierarchical promotion and long-term stability, today’s flexible, individual-preference-based and personal development-centered career approaches come to the fore (Huang et al., 2022).

Career satisfaction is defined in the most general approach as “the sum of the positive psychological outcomes that an individual obtains as a result of their work experiences” (Khattak et al., 2022). In other words, career satisfaction is a multidimensional, dynamic concept that includes a process in which an individual evaluates their perception of their career, questions the harmony between their expectations and the experiences they have gained, and expresses the level of satisfaction in their professional lives (Hagmaier et al., 2018). The process of evaluating an individual’s career satisfaction consists of various cognitive and emotional stages. First, the individual determines their professional goals and expectations. These goals are shaped by personal values, abilities, and professional interests (Xi et al., 2022). Then, the individual makes an evaluation by comparing the current career situation with these expectations. If the individual’s career progresses in line with the goals and expectations, he/she has set, this situation results in high satisfaction; while deviations from goals or failure to meet expectations may lead to dissatisfaction (Wu et al., 2024; Pajo, 2025). During the evaluation process, the individual considers elements such as working conditions, career development opportunities, work-life balance, and leadership practices. Employees’ intrinsic and extrinsic motivations for their careers are among the important factors that determine the level of satisfaction (Lent and Brown, 2020). In the context of intrinsic motivation, the individual also evaluates his/her self-efficacy perception regarding his/her career throughout the process and predicts his/her future professional development. This process is dynamic and may vary depending on the changes, new opportunities, and organizational factors experienced by the individual in his/her career. In terms of extrinsic motivation, factors such as the experiences employees gain during their careers, professional development opportunities and work-life balance play a decisive role in the formation of this satisfaction (Pajo, 2024).

While traditional approaches associate career satisfaction with job satisfaction, today it is accepted that this concept has a broader perspective and is linked to the individual’s professional identity, subjective perception of success and long-term professional satisfaction (Singh, 2018). However, career satisfaction is not limited to individuals’ personal experiences but is also of critical importance for organizations. When evaluated from the perspective of employees, high career satisfaction increases the individual’s commitment to their job, motivation, performance and general life satisfaction (Gerçek et al., 2024). Individuals who are satisfied with their careers tend to be more willing, productive and innovative in achieving their professional goals. In addition, low career satisfaction has been associated with negative outcomes such as burnout syndrome, stress and intention to leave the job (Lent and Brown, 2020). From the perspective of organizations, employees’ career satisfaction is an important factor that increases productivity in the workplace, strengthens organizational commitment and retains talented employees (Eddleston, 2009). In organizations where career satisfaction is high, employees are observed to be more proactive, job satisfaction increases, and turnover rates decrease (Aburumman et al., 2020). Organizations can develop strategies to meet career expectations by offering career development opportunities to their employees and creating training programs and mentoring systems. In addition, a fair promotion process, qualified leadership practices, performance-based reward systems, and work-life balance policies are among the important practices that positively affect employees’ career satisfaction (Yao and Ma, 2024).

## 3 The relationship between concepts

With globalization and increasing competition, businesses are turning to digital transformation in order to provide sustainable competitive advantage. The need for a new leadership approach that will guide digital transformation is emerging in this respect. In this respect, it can be said that digital leadership has become a phenomenon (Sagbas et al., 2023). Digital leadership is seen as a more comprehensive type of leadership that includes transformational leadership. In this context, digital leaders are expected to play a proactive role in achieving organizational goals and objectives, motivate employees, and encourage innovative and creative ideas (Peter et al., 2020). How the expectations will be met by the digital leader and the underlying reasons are still not fully explained in the literature. In this respect, this study contributes to the literature in explaining how the leader motivates the employees and its effect on HWI together with Self-Determination Theory (Deci and Ryan, 1985). In Self-Determination Theory (SDT), there are three basic elements for employee motivation: autonomy, competence and relatedness. The degree to which these three elements are satisfied helps to explain the social environment, behavior and how employees are motivated (van Beek et al., 2012). When these essential elements are met, employees experience higher motivation and well-being, and when they are disappointed, they face disconnection and job strain (Costantini et al., 2025). In terms of motivating employees, a binary distinction is made between intrinsic and extrinsic motivation. Intrinsic motivation refers to performing an action because the employee experiences it as inherently enjoyable, interesting and challenging. Extrinsic motivation is created by external conditions, including external behavior, punishment, threat, or material and social rewards (Taris et al., 2020). Within the scope of studies examining the current impact of SDT on HWI, it is seen that workaholic employees work hard to maintain and improve their feelings of self-worth and self-esteem and because they personally care about the associated outcomes. It has also been found that engaged employees work hard because they tend to experience work activities as interesting, enjoyable and satisfying (Houlfort et al., 2014). In this respect, it is thought that it is possible for the leader to direct employees to invest heavily in work and to combine organizational goals with employee goals.

Evaluating the interaction between the digital leader and HWI only in the context of SDT would be shallow and mechanical. For this reason, it is necessary to remember that human beings and organizations are social structures and that there is social change within them. It is seen that employees act in accordance with the social change that they will gain (career, salary, etc.) in the organization and stay away from initiatives that will cause harm. In this respect, it is thought that the digital leader can create heavy work investors within the organization by taking into account the needs of the employees (Čerović et al., 2020). Social Exchange Theory (SET) states that when the organization shows a positive attitude toward the employee, the employee will show a positive attitude toward the organization (Blau, 1964). It is seen in the literature that it is possible to establish an incentive system for heavy work investment by motivating employees based on the theory (Rabenu and Shkoler, 2022). In short, the theory explains the constructive social exchange and interaction between the digital leader and the employee based on mutual trust, reciprocity and fairness expectations and encourages employees for heavy work investment (Tabak et al., 2021).

Digital leaders can manage their work by choosing remote workplaces and establishing virtual teams to complete tasks independently of a specific time and place, as well as changing work demands in general (Zhu et al., 2022). In digital leadership, the establishment of virtual teams is felt as a necessity and it is seen that there is a continuous management philosophy without time limitations. In this philosophy, the work priorities are time, reliability, quality and cost, respectively, and it turns into an environment where dynamic participation is experienced (El Sawy et al., 2016). Based on this, it is known that there are attempts to create a structure where time is important, and customer needs are met in the shortest time. Since there is no previous study that evaluates digital leadership and HWI together, it is thought that digital leadership will have an impact on HWI as a result of the understanding that job demands and job resources will be balanced within the scope of JD-R Theory, that digital leaders will motivate employees to invest heavily in HWI within the scope of SDT, and the development of a mutual, win-win relationship with SET. Therefore, H1 and H2 hypotheses were formed.

Based on this, H_1_ and H_2_ hypotheses were created.

*H_1_.* Digital leadership has a significant effect on TC.

*H_2_.* Digital leadership has a significant effect on WI.

It is seen that digital leadership has a significant effect on job performance, and the effect stated in the literature is explained by Upper Echelon, Social Exchange Theory, JD-R Theory and RBV Theory (Mihardjo et al., 2019a; Abbu et al., 2022; Benitez et al., 2022; Sagbas et al., 2023; Topcuoglu et al., 2023a). In the study conducted by Sagbas et al. (2023) on 390 employees, a low-level effect was determined, and this effect was associated with the transformational, innovative, communicative and supportive attitude of a digital leader motivating his employees to exhibit higher performance. In the study conducted by Topcuoglu et al. (2023a) on 308 employees, a low-level effect was determined, and the result was associated with the effect of the leader’s past experiences and current abilities on the employees. Based on this, the H_3_ hypothesis was created.

*H_3_.* Digital leadership has a significant effect on job performance.

It is seen that digital leadership has an effect on job satisfaction, and the effect stated in the literature is explained by JD-R Theory and RBV Theory (Sulistiana and Darma, 2023; Topcuoglu et al., 2023a). In the study conducted by Topcuoglu et al. (2023b) on 403 employees, a low-level effect was determined, and this effect was associated with digital leadership taking the necessary precautions to protect valuable and inimitable human resources. In the study conducted by Sulistiana and Darma (2023) on 207 employees, a low-level effect was determined, and the result obtained was associated with the higher the digital leadership perceptions felt by the employees, the higher the job satisfaction of the employees. Based on this, the H_4_ hypothesis was created.

*H_4_.* Digital leadership has a significant effect on job satisfaction.

Digital leadership brings about the formation of new organizational units and new career opportunities in the digital transformation phase. It is seen that career development is organized in accordance with the implementation of new strategies and needs assessment (Shah, 2020). When the studies are examined, it is seen that the works related to career are generally compilation articles based on theoretical foundations and there are no works containing research (Zia et al., 2024). In this respect, inspired by the RBV Theory (Barney, 2001), it is thought that digital leaders will guide employees for a new career construction in the ownership of big data analytical capabilities, material resources, non-material resources, human skills and knowledge (Wang et al., 2022). RBV Theory is an important theory that explains how organizations provide sustainable competitive advantage through the resources they have and is frequently used in the field of human resources (Mihardjo et al., 2019b). Based on this, the H_5_ hypothesis was created.

*H_5_.* Digital leadership has a significant effect on career satisfaction.

Van Beek et al. (2014) demonstrated a strong interaction between HWI variables and job satisfaction and job performance. Similarly, a strong interaction between HWI variables and job satisfaction and job performance was determined in a study conducted by Bocean et al. (2020) on 298 employees in Romania. A strong interaction between HWI variables and job satisfaction was determined in a study conducted by Nemteanu and Dabija (2020) on 766 people in Romania. In a study conducted by Popa et al. (2020) with a total of 822 employees in Japan and Romania, it was determined that HWI-TC had an effect on job performance (*ß* = −0.093; *p* < 0.05), HWI-TC had an effect on job satisfaction (*ß* = −0.117; *p* < 0.05), HWI-WI had an effect on job performance (*ß* = 0.370; *p* < 0.05), and HWI-WI had an effect on job satisfaction (*ß* = 0.386; *p* < 0.05). In the literature, it is stated that one of the aims of those who make HWI is to achieve career development (Houlfort et al., 2014; Snir, 2018; Butnaru et al., 2020). In the study conducted by Kaygin et al. (2023) with 362 public employees in Turkiye, it is seen that there is a low level of interaction between HWI variables and career. In this context, the following hypotheses have been developed.

*H_6_.* TC has a significant effect on job performance.

*H_7_.* TC has a significant effect on job satisfaction.

*H_8_.* TC has a significant effect on career satisfaction.

*H_9_.* WI has a significant effect on job performance.

*H_10_.* WI has a significant effect on job satisfaction.

*H_11_*. WI has a significant effect on career satisfaction.

Digital leadership requires the leader to develop, manage, direct and implement information technologies to improve organizational performance (AlAjmi, 2022). Digital leaders usually manage their work within a digital structure or through a platform (Benitez et al., 2022). In this way, employees’ instant production and control of their work are also provided, and instant job analysis is also provided with artificial intelligence technologies. It is seen that digital leadership is modestly related to employees and motivates them in these processes (Khaw et al., 2022). In addition, strong social networks, digital skills, cooperation, participation and visionary nature of digital leadership come to the fore (Erhan et al., 2022). In the light of these criteria, it is thought that digital leadership will act in accordance with job demands and resources, motivate and direct employees, inspired by the JD-R theory. In this context, the following hypotheses have been developed.

*H_12_.* TC has a mediating role in the effect of digital leadership on job performance.

*H_13_.* TC has a mediating role in the effect of digital leadership on job satisfaction.

*H_14_.* TC has a mediating role in the effect of digital leadership on career satisfaction.

*H_15_.* WI has a mediating role in the effect of digital leadership on job performance.

*H_16_.* WI has a mediating role in the effect of digital leadership on job satisfaction.

*H_17_.* WI has a mediating role in the effect of digital leadership on career satisfaction.

**Figure 1:**
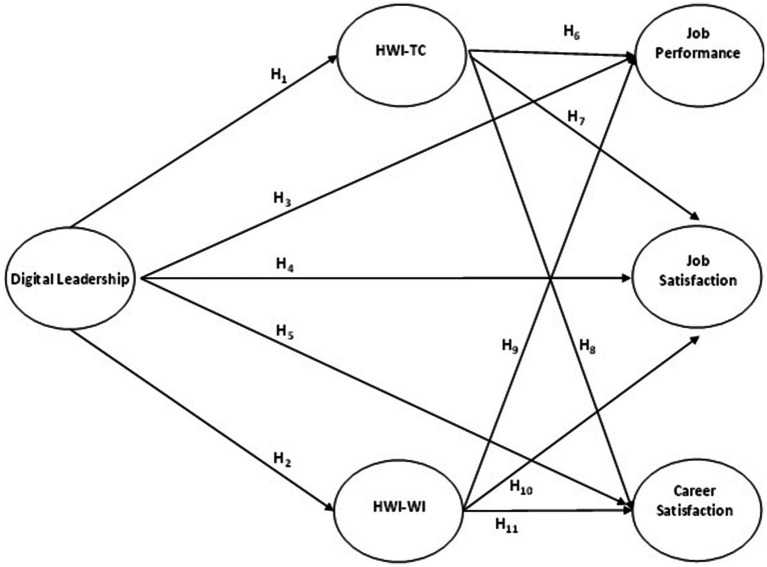
Research model.

The working model for the hypotheses created is presented in Figure 1.

## 4 Method

The universe of this study consists of employees of small and medium-sized enterprises operating in the field of information technologies in Adana province. According to the Turkish Statistical Institute, SMEs are defined as enterprises with less than 250 employees and whose balance sheet does not exceed 250 million TL. They constitute 99.7% of all enterprises in Turkiye and contribute 36.4% to the added value (Turkstat, 2023). According to the Turkish Statistical Institute, approximately 2 million 300 thousand people live in Adana (Turkstat, 2024). Data was collected from 43 different SMEs in Adana, and it is possible to talk about a sample of approximately 20,000 people.

The formula suggested by Cochran (1977) in Equation 1 was used to determine the minimum number of participants that would represent the sample in question. In accordance with the literature, a confidence level of 95% and a margin of error of 5% were used to ensure the statistical reliability and generalizability of the findings (Uygungil-Erdogan et al., 2025). The expression n in the formula represents the population size, the expression p represents the percentage of occurrence of a situation or condition, the expression e represents the margin of error, and the expression z represents the confidence level (1.96% at a 95% confidence interval) (Alzoubi et al., 2024).


(1) $$n_{0}=\frac{Z^{2}pq}{e^{2}}$$

In this context, at least 377 people need to be reached with the calculation made according to the formula. Three hundred and ninety three people were reached in the study using the convenience sampling method. Convenience sampling provides effective data collection by providing easy access to the people participating in the sample. Since convenience sampling is based on voluntary participation, it is a method that is likely to include people with strong knowledge and feelings about the subject (Andrade, 2021). However, the fact that the study data were obtained from SMEs located in only one province in Turkiye with a cross-sectional approach constitutes an important limitation in terms of generalizability of the findings. The data obtained as a result of the survey were analyzed with Smart-PLS version 3.2.9. This application was preferred due to its suitability for evaluating models with a large number of components and indicators. In this context, Smart-PLS is a frequently preferred method due to the simultaneous estimation of multiple and interrelated dependent relationships between variables and the simultaneous measurement of latent structures (Kaya et al., 2020). The data obtained from the participants are processed into the Smart-PLS program, and the findings are obtained by performing PLS Algorithm and Bootstrapping calculations on the program. Before the survey application, permission was obtained from Giresun University Ethics Committee with the letter numbered 11/28 dated 04 December 2024.

All scales used in data collection used a 5-point Likert-type scale (Likert, 1932) with responses ranging from 1 = Strongly Disagree to 5 = Strongly Agree. The Digital Leadership Scale, developed by Zeike et al. (2019) and adapted to Turkish by Oktaysoy et al. (2022), was used to measure Digital Leadership. The scale consists of six statements and a single dimension. The Cronbach Alpha value of the original scale was determined as 0.870. The scale includes statements such as “I think using digital tools is fun.”

The Effort Scale developed by Brown and Leigh (1996) was used to measure HWI. The scale consists of ten statements and two dimensions. The Cronbach Alpha value of the original scale was determined as 0.860 for the TC sub-dimension and 0.820 for the WI dimension. The scale includes statements such as “Other people know me by the long hours I keep.”

The Job Performance Scale developed by Sigler and Pearson (2000) and adapted to Turkish by Ay and Keleş (2017) was used to measure job performance. The scale consists of four statements and one dimension. The Cronbach Alpha value of the original scale was determined as 0.830. The scale includes statements such as “I have mastered the skills necessary to do my job.”

The Job Satisfaction Scale developed by Judge et al. (1998) and adapted into Turkish by Başol and Çömlekçi̇ (2020) was used to measure job satisfaction. The scale consists of five statements and a single dimension. The Cronbach Alpha value of the original scale was determined as 0.890. The scale includes statements such as “‘I feel fairly well satisfied with my present job.”

The Career Satisfaction Scale developed by Greenhaus et al. (1990) and adapted into Turkish by Avcı and Turunç (2012) was used to measure career satisfaction. The scale consists of five statements and a single dimension. The Cronbach Alpha value of the original scale was determined as 0.880. The scale includes statements such as “I am satisfied with the success I have achieved in my career.”

## 5 Findings

In the study, 393 people were reached and detailed socio-demographic information about the individuals who participated in the study is presented in Table 2. More than half of the participants (54.7%) were male, and the rest (45.3%) were female. In general, the prevalence of female employment stands out compared to the conditions in Turkiye, where male employment is higher. Especially in the IT sector, it is desired for customer representatives and accounting officers to be women. Culturally, women are treated more politely and are more hesitant in bargaining (Aydın and Görgülü, 2023). It is seen that the number of married participants (65.4%) is higher than single participants. It was found that most of the participants (61.6%) had a bachelor’s degree and the vast majority (63.1%) had 6–10 years of experience. It is also seen that all participants receive a wage higher than the minimum wage applied in Turkiye.

**Table 2:** Demographic information.

Demographic	Variable	*n*	%
Gender	Female	178	45.30
	Male	215	54.70
Marital Status	Married	257	65.40
	Single	136	34.60
Age	Between 18–30 Years Old	42	10.70
	Between 31–40 Years Old	268	68.20
	Between 41–50 Years Old	58	14.70
	51 Years Old and Over	25	6.40
Level of Education	High School	39	9.90
	Associate degree	84	21.40
	Bachelor’s degree	242	61.60
	Postgraduate Degree	28	7.10
Sectoral experience	5 Years and Below	27	6.90
	Between 6–10 Years	248	63.10
	Between 11–15 Years	59	15.00
	Between 16–20 Years	38	9.70
	21 Years and Above	21	5.30
Income	Between 40.000–50.000 Turkish Lira	38	9.70
	Between 50.001–60.000 Turkish Lira	102	26.00
	Between 60.001–70.000 Turkish Lira	113	28.70
	Between 70.001–80.000 Turkish Lira	68	17.30
	80.001 Turkish Lira and Above	72	18.30

In terms of validity and reliability of the study, some values stand out. In this respect, factor loading values are greater than 0.50, Cronbach Alpha, Composite Reliability and rho_A coefficients are greater than 0.70 and Average Variance Extracted (AVE) value is greater than 0.50, which is an important sign in terms of ensuring validity and reliability (Hair et al., 2014; Sarstedt et al., 2022). When the values of the scales in Table 3 are examined, it is seen that internal consistency and convergent validity are provided because all structures are above the threshold values (Oktaysoy et al., 2025).

**Table 3:** Factor load values, validity and reliability.

Item	Factor loading	Median	Standard deviation	Kurtosis	Skewness
Digital leadership scale					
Cronbach’s alpha = 0.886, rho_A = 0.896, CR = 0.914, AVE = 0.640					
DIG1	0.673	3.667	1.109	−0.302	−0.717
DIG2	0.838	3.445	1.018	−0.986	−0.178
DIG3	0.839	3.616	1.076	−0.371	−0.665
DIG4	0.785	2.990	1.189	−1.115	0.083
DIG5	0.809	3.384	1.106	−0.695	−0.531
DIG6	0.843	3.277	1.049	−0.717	−0.255
**HWI-TC scale**					
Cronbach’s Alpha = 0.887, rho_A = 0.893, CR = 0.917, AVE = 0.690					
HWITC1	0.823	3.351	1.176	−0.947	−0.323
HWITC2	0.864	3.310	1.164	−0.865	−0.304
HWITC3	0.863	3.216	1.190	−0.969	−0.197
HWITC4	0.824	3.282	1.152	−1.011	−0.117
HWITC5	0.775	3.112	1.099	−0.843	−0.050
**HWI-WI scale**					
Cronbach’s Alpha = 0.914, rho_A = 0.931, CR = 0.935, AVE = 0.743					
HWIWI1	0.792	4.041	0.828	2.239	−1.211
HWIWI2	0.870	4.168	0.793	4.300	−1.602
HWIWI3	0.891	4.122	0.769	3.023	−1.259
HWIWI4	0.894	4.361	0.753	4.175	−1.640
HWIWI5	0.857	4.158	0.865	2.332	−1.304
**Job performance scale**					
Cronbach’s Alpha = 0.869, rho_A = 0.877, CR = 0.910, AVE = 0.718					
PERF1	0.848	4.201	0.747	2.520	−1.155
PERF2	0.817	4.115	0.810	1.631	−1.077
PERF3	0.859	4.153	0.704	0.918	−0.663
PERF4	0.864	4.237	0.671	1.516	−0.776
**Job satisfaction scale**					
Cronbach’s Alpha = 0.912, rho_A = 0.958, CR = 0.932, AVE = 0.735					
SATICF1	0.841	4.242	0.868	2.415	−1.404
SATICF2	0.779	3.621	0.999	−0.220	−0.428
SATICF3	0.885	3.967	0.966	0.423	−0.886
SATICF4	0.888	3.980	0.933	0.793	−0.961
SATICF5	0.887	3.901	0.934	0.409	−0.815
**Career satisfaction scale**					
Cronbach’s Alpha = 0.921, rho_A = 0.932, CR = 0.941, AVE = 0.762					
CAREER1	0.890	3.817	1.030	0.032	−0.792
CAREER2	0.924	3.799	0.985	0.262	−0.776
CAREER3	0.780	3.501	1.101	−0.730	−0.388
CAREER4	0.920	3.786	0.965	0.657	−0.891
CAREER5	0.842	3.814	0.970	0.582	−0.882

Common method bias, arising from the method used to collect data, often refers to concerns about artificially inflated relationships between variables. Since the Variance Inflation Factor (VIF) value of the expressions used in the study was below 10, it was determined that there was no common method bias for the expressions (O’brien, 2007). With the increasing criticism on VIF values, marker-based techniques have started to be proposed for the detection of common method bias (Simmering et al., 2015). In this study, the marker variable technique was applied over HWI-TC for the detection of common method bias and it was determined again that there was no common method bias in the study. When the obtained results were evaluated in the light of the threshold values, it was understood that the scales did not have multi-collinearity and common method bias. It is not enough for the mandatory tests specified as validity, reliability and common method bias to be suitable for the research, and discriminant validity analysis, which allows the scales to be distinguished from each other, should also be performed (Hair et al., 2017). Discriminant validity is a criterion used to determine the extent to which variables in a scale are distinguished from variables in other scales (Sarstedt et al., 2022). When the literature is examined, the criteria suggested by Fornell and Larcker (1981) and Henseler et al. (2015) are known as the most frequently used discriminant validity method (Hair et al., 2017). Discriminant validity values according to Fornell and Larcker and Heterotrait-Monotrait criteria are shown in Table 4.

**Table 4:** Discriminant validity.

Item	**Fornell-Larcker Criterion and Heterotrait-Monotrait Ratio (HTMT)**					
	1	2	3	4	5	6
Career satisfaction	0.873	0.341*	0.186*	0.352*	0.744*	0.458*
Digital leadership	0.306	0.800	0.262*	0.346*	0.273*	0.333*
HWI-TC	0.168	0.233	0.831	0.478*	0.156*	0.407*
HWI-WI	0.339	0.324	0.423	0.862	0.343*	0.521*
Job satisfaction	0.681	0.260	0.145	0.347	0.857	0.548*
Job performance	0.414	0.301	0.367	0.475	0.520	0.847

Values marked with * belong to HTMT analysis.

The test performed is important in terms of evaluating whether there is a high overlap between the measurement variables of a model and whether the distinction between the structures is sufficient (Atan and Obeng, 2024; Galanis et al., 2024). In the Fornell-Larcker Criterion, the square roots of the AVE coefficients are used to ensure discriminant validity (Fornell and Larcker, 1981). According to the Heterotrait-Monotrait criteria, the correlation threshold value should be below 0.90 (Henseler et al., 2015). The values obtained were significantly below the specified threshold for all variables; this shows that each scale structure is different and separate (Uygungil-Erdogan et al., 2025).

**Figure 2:**
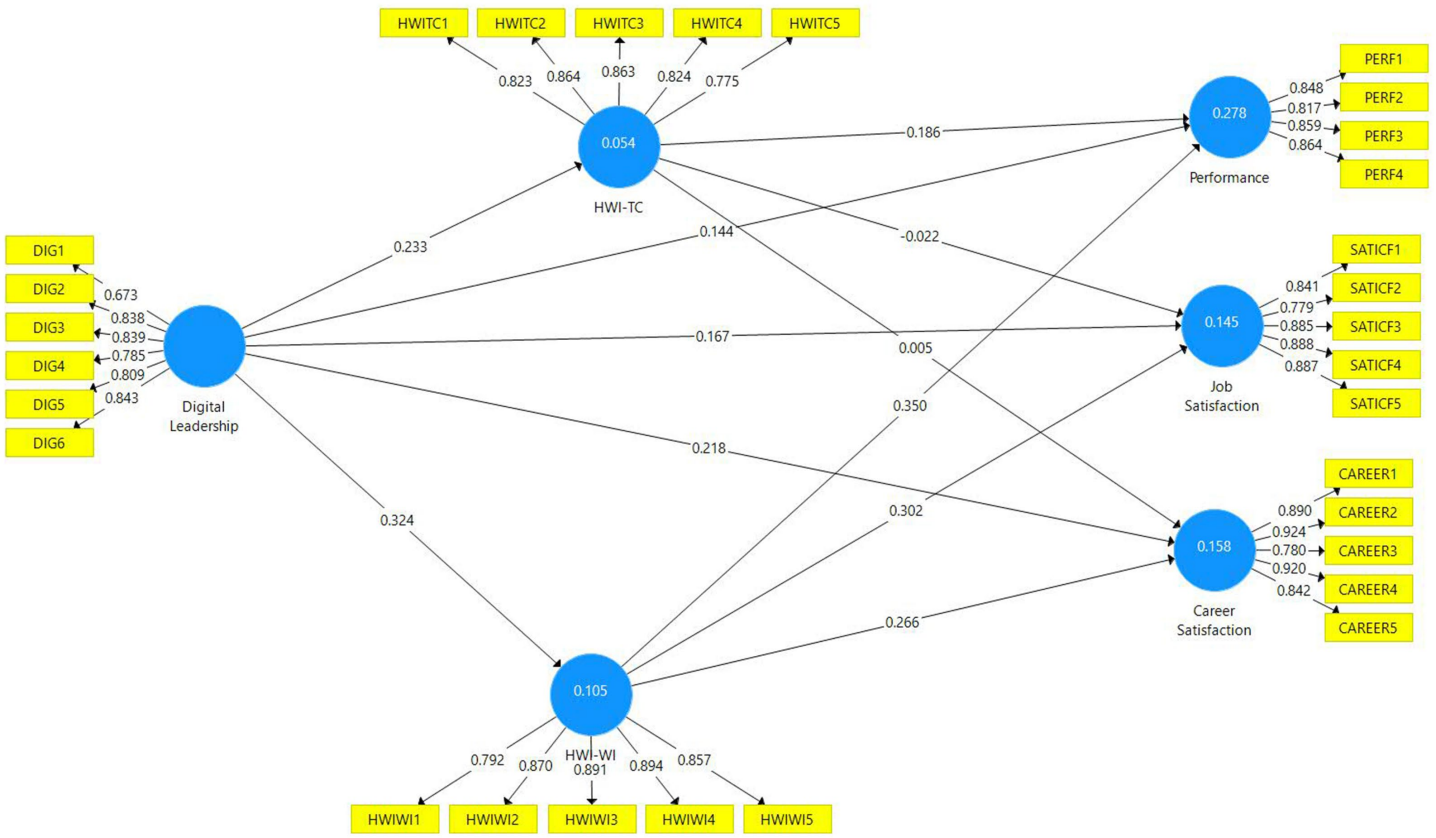
Research model path diagram.

In order to test the model in the study, a mediation analysis was performed by choosing a bootstrapping sampling number of 5.000 in the Smart-PLS program with the “Bootstrapping” method. In the bootstrap test developed by Efron (1979), the sample number of 393 in the study is randomly increased to 5.000 through the Smart-PLS program and the analysis is performed. The bootstrapping method helps to accurately estimate standard errors and confidence intervals for path coefficients. At the same time, the method provides a reliable basis for hypothesis testing (Hair et al., 2017). As a result of the test, beta, p and t values were examined to test whether the path coefficients were statistically significant (Sarstedt et al., 2022). Figure 2 shows the Smart-PLS diagram obtained from the research model.

The structural model is shown in Figure 2, and when the goodness of fit values are examined, the SRMR<0.080 value is determined as 0.061, the d_ULS value as 1.729 and the d_G value as 0.719. The Chi-Square <5 value is found as 1.630 and the NFI > 0.80 value as 0.820. In this context, it is seen that the goodness of fit values obtained from the model are within acceptable limits. When the relevant values are examined, it is recommended that the Standardized Root Mean Square Residual (SRMR) value be below 0.08 and the Normed Fit Index (NFI) value be above 0.80 (Byrne, 2016). Since the obtained results are above the threshold values, the model provides the goodness of fit values and hypothesis testing is carried out. The values related to the hypothesis tests are presented in Table 5. Regarding hypothesis testing, t values above 1.96 and *p-*values below 0.05 at 95% confidence level and 5% margin of error indicate that the hypothesis is significant (Hair et al., 2017). ß values below 0.29 are interpreted as low effects, effect sizes between 0.30–0.49 as medium effects, and effect sizes of 0.50 or more as high effects (Nieminen, 2022).

**Table 5:** Hypothesis test result.

Path analysis	Estimate	Standard deviation	*t*-values	*p*	VAF values	Support
Digital Leadership - > HWI-TC	0.233	0.056	4.195	0.000		H1 accept
Digital Leadership - > HWI-WI	0.324	0.045	7.217	0.000		H2 accept
Digital Leadership - > Job Performance	0.144	0.046	3.106	0.002		H3 accept
Digital Leadership - > Job Satisfaction	0.167	0.054	3.093	0.002		H4 accept
Digital Leadership - > Career Satisfaction	0.218	0.053	4.132	0.000		H5 accept
HWI-TC - > Job Performance	0.186	0.050	3.713	0.000		H6 accept
HWI-TC - > Job Satisfaction	−0.022	0.058	0.371	0.711		H7 reject
HWI-TC - > Career Satisfaction	0.005	0.055	0.092	0.927		H8 reject
HWI-WI - > Job Performance	0.350	0.083	4.223	0.000		H9 accept
HWI-WI - > Job Satisfaction	0.302	0.062	4.876	0.000		H10 accept
HWI-WI - > Career Satisfaction	0.266	0.058	4.600	0.000		H11 accept
Digital Leadership - > HWI-TC - > Job Performance	0.043	0.016	2.673	0.008	0.231	H12 accept partial
Digital Leadership - > HWI-TC - > Job Satisfaction	−0.005	0.014	0.354	0.724	–	H13 reject
Digital Leadership - > HWI-TC - > Career Satisfaction	0.001	0.014	0.087	0.931	–	H14 reject
Digital Leadership - > HWI-WI - > Job Performance	0.113	0.030	3.843	0.000	0.441	H15 accept partial
Digital Leadership - > HWI-WI - > Job Satisfaction	0.098	0.023	4.342	0.000	0.369	H16 accept partial
Digital Leadership - > HWI-WI - > Career Satisfaction	0.086	0.020	4.212	0.000	0.283	H17 accept partial

When Table 5 is examined, it is seen that digital leadership has a positive and significant effect on TC (*ß* = 0.233; *p* < 0.05), WI (*ß* = 0.266; *p* < 0.05), job performance (*ß* = 0.144; *p* < 0.05), job satisfaction (*ß* = 0.167; *p* < 0.05) and career satisfaction (*ß* = 0.218; *p* < 0.05). Based on this, hypotheses H1, H2, H3, H4, and H5 are accepted. TC has a positive and significant effect on job performance (*ß* = 0.144; *p* < 0.05), and it has no significant effect on job satisfaction (*ß* = −0.022; *p* > 0.05), and career satisfaction (*ß* = 0.005; *p* > 0.05). Based on this, H6 was accepted and hypotheses H7 and H8 were rejected. WI has a positive and significant effect on job performance (*ß* = 0.350; *p* < 0.05), job satisfaction (*ß* = 0.302; *p* < 0.05) and career satisfaction (*ß* = 0.266; *p* < 0.05). Based on this, hypotheses H9, H10, and H11 were accepted. It was determined that TC has a mediating role in the effect of digital leadership on job performance (*ß* = 0.043; *p* < 0.05), and hypothesis H12 was accepted. It was determined that TC did not have a mediating role in the effect of digital leadership on job satisfaction (*ß* = −0.005; *p* > 0.05), and hypothesis H13 was rejected. It was determined that TC did not have a mediating role in the effect of digital leadership on career satisfaction (*ß* = 0.001; *p* > 0.05), and hypothesis H14 was rejected. It was determined that WI had a mediating role in the effect of digital leadership on job performance (*ß* = 0.113; *p* < 0.05), and hypothesis H15 was accepted. It was determined that WI had a mediating role in the effect of digital leadership on job satisfaction (*ß* = 0.113; *p* < 0.05), and hypothesis H16 was accepted. It was determined that WI had a mediating role in the effect of digital leadership on career satisfaction (*ß* = 0.113; *p* < 0.05), and hypothesis H17 was accepted. With the acceptance of the hypotheses regarding mediation, the significance of the Variance Accounted For (VAF) value of the hypotheses should also be examined. In this respect, in the calculation made with VAF, it is stated that the VAF value is 0–20% no mediation, 20–80% partial mediation, 80–100% full mediation (Henseler et al., 2015). It was determined that all of the hypotheses accepted to have a mediation relationship showed partial mediation.

## 6 Conclusion and discussion

Today, the effects of digital leadership on employee motivation and performance are increasingly being investigated in the business world. However, there are limited studies on the mechanisms through which these effects occur and what role HWI plays in this process. Based on this point, this research examines the effects of digital leadership on employees’ job performance, job satisfaction and career satisfaction, and evaluates the mediating role of the concept of HWI in this relationship. The research, which was conducted on the basis of JD-R Theory, revealed how the balance between job demands and resources shapes employees’ performance and satisfaction levels (Demerouti, 2018). The sample of the study consists of 393 people working in SMEs operating in the information technologies sector in Adana, Turkiye. The participants were selected by convenience sampling method and consist of individuals working in different positions. The data were analyzed using the Smart-PLS application.

The analyses show that digital leadership has a positive and significant effect on employees’ job performance, job satisfaction, and career satisfaction. Digital leaders’ motivating, supportive, and technology-focused management approach contributes to employees developing a higher sense of commitment and satisfaction in their work processes. This finding is consistent with previous studies emphasizing that digital leadership is a factor that increases employee commitment and performance (Benitez et al., 2022; Sagbas et al., 2023).

When the mediating role of HWI was examined, it was determined that the WI dimension showed a partial mediating role in the relationship between digital leadership and job performance, job satisfaction, and career satisfaction. This situation reveals that employees can be more satisfied and perform better when they adapt to a high work tempo, but this effect needs to be supported by job resources to be sustainable (Schaufeli, 2016; Snir and Harpaz, 2021). On the other hand, it was determined that the TC dimension did not have a significant mediating role on job satisfaction and career satisfaction. This finding indicates that employees who invest excessive time in their jobs may encounter negative consequences such as burnout and stress instead of being satisfied with their jobs (Tziner et al., 2019). When this finding is evaluated within the framework of JD-R theory, it can be said that when job demands are excessively high and job resources are insufficient, the likelihood of employees experiencing loss of motivation and burnout increases (Rattrie et al., 2020). In addition, current developments have a significant impact on employees’ tendency toward negative work behaviors. For example, with the participation of Generation Z in business life, expectations for flexible working have increased, and it is seen that the interest in working 4 days a week and reducing working hours has increased (Johns Hopkins University, 2023). In another example, during the Covid-19 pandemic, which deeply affected working life, flexible working became widespread, and the importance of physical offices decreased with the increase in Zoom-like applications for the ability to work remotely (Kaygın and Topçuoğlu, 2020). In this respect, not meeting the expectations of employees reveals stress, tension and burnout. Apart from international developments, it is seen that some practices in Türkiye cause negative work behaviors. For example, employees who work overtime in Türkiye have serious problems in receiving their wages. According to a study conducted in 2024, it was determined that only half of the employees who worked overtime could receive their wages (Akça, 2024). Therefore, the fact that the mediating effect of HWI-TC is insignificant for some variables is related to the Social Exchange Theory. Failure to meet the demands of employees resulted in employees not spending more time at the workplace.

## 7 Managerial/practical implications

The research results reveal that organizations should consider the work-investment balance of employees when determining digital leadership strategies. Developing strategies that increase work commitment and prevent burnout is a critical point in terms of protecting employee well-being. Digital leaders encourage their employees to invest more in work while also considering their work-life balance will be a factor that supports organizational sustainability in the long term (Snir and Harpaz, 2012). At this point, the following inferences can be made regarding managerial processes based on the research findings.

- Digital leaders should develop strategies to increase employee engagement. Making business processes transparent, providing open communication and supporting employees’ adaptation to digital transformation will play an important role in this process.
- The long-term negative effects of excessive working hours and workaholism should be taken into consideration. Employees should be provided with a balanced work-life relationship, and excessive workload should be managed in a way that does not lead to burnout.
- Digital leadership practices should be planned to support employees’ career development. Increasing technological skills, providing training opportunities and supporting employees’ long-term career goals will contribute to increasing career satisfaction.
- Workload should be balanced for employees who work TC, and mechanisms should be created to prevent long working hours from causing burnout.
- Considering the positive effects of the WI dimension, flexible working models that enable employees to work efficiently and focus should be encouraged.
- In addition to guiding technological transformation, digital leaders must be able to establish a balance at this point by considering the psychological and physical boundaries of employees.

There are serious studies showing that HWI can cause health problems for employees (Snir and Harpaz, 2021; Snir et al., 2023). In addition, measures need to be taken against potential threats such as burnout, work-life imbalance and digital fatigue that digital leadership will create in the organization. In general, studies do not explain why negative work behaviors such as burnout are undesirable, but it is seen that the reader is expected to make sense of it. Studies have shown that in the United States of America, overwork, ineffectiveness and burnout of employees for a year costs the employer between 3,999 dollars and 20,683 dollars per year depending on the position of the average employee. For an organization with a total of one thousand employees, the loss due to negative work behaviors is expected to be approximately 5.04 million dollars annually (Martinez et al., 2025). Due to the high costs of negative work behaviors, many measures are taken to prevent them. In this respect, targeted strategies are proposed to increase leaders’ ability to manage technological demands and effectively manage digital transformations. With digitalization, organizations need to re-evaluate their operational methods and integrate digital technologies into their daily processes. Therefore, organizations need to strategize on methods that can encourage employees to voluntarily adopt digital knowledge and technology to gain proficiency in digital skills (Chen and Zhao, 2024). In this respect, it is considered useful to train leaders in terms of creativity, thinking and questioning, global vision and collaboration, discovery and deep knowledge (Li et al., 2025).

## 8 Theoretical implications

With the help of this study, the mediating role of heavy work investment in the effect of digital leadership on employees’ job performance, job satisfaction and career satisfaction is tried to be explained with various theories. The interaction between digital leadership and heavy work investment is associated with JD-R, SDT and SET theories. These theories explain the relationship between the two variables through motivation, mutual exchange and demand-resource relationship. In short, these theories indicate that the leader should have sufficient resources, a suitable environment for motivation (autonomy, competence and a relevant organizational structure), and an organizational structure that develops on mutual trust, reciprocity and justice expectations between the leader and the employee. Therefore, while the mediating role of HWI-WI is determined, albeit weakly, it is seen that HWI-TC does not have a significant mediating role especially on job satisfaction and career satisfaction variables in Türkiye, where overtime wages are a problem. The obtained result shows that all three theories support the hypotheses, albeit at a low level. In this context, it can be said that a contribution has been made to a small number of HWI researches in the field of digital leadership. The low level effect of digital leadership on job performance, job satisfaction and career satisfaction is associated with RBV, JD-R and SET theories. It is seen that these theories are frequently referred to in the literature (Sagbas et al., 2023; Topcuoglu et al., 2023a, 2023b). The low-level effect of heavy work investment on job performance, job satisfaction and career satisfaction is associated with the JD-R theory (Tziner et al., 2019). In this context, it is seen that a known situation in the literature is repeated.

## 9 Limitations and directions for future research

This study, while making significant contributions to literature, also has some limitations in terms of scope and methodology. The first of these limitations is the sample limitation. The study was limited to SME employees operating in the IT sector in Turkiye. This may limit the generalizability of the findings to different sectors or large-scale enterprises. Another limitation is related to cultural differences. Considering that the concepts of digital leadership and heavy-duty investment may have different meanings in a cultural context, conducting the study only in Turkiye creates limitations in terms of whether similar results can be obtained in different cultural environments.

Considering the limitations listed above, some suggestions can be made for researchers for further studies. The first of these is the suggestion that the research be applied to different sectors and lines of business. In order to increase the generalizability of the findings, similar studies should be conducted on employees in different sectors. The effects of digital leadership on employees should be examined in detail, especially in areas where digital transformation processes can have different effects, such as manufacturing, health and education. Another suggestion is to conduct qualitative research instead of quantitative research. Qualitative research methods can be effective in order to understand employees’ perceptions of digital leadership, their attitudes toward HWI and their work-life balance more deeply. Another suggestion is to conduct comparative studies focusing on cultural differences. In order to compare the effects of the concepts discussed in research in different countries, comparative studies to be conducted in different cultural contexts are very important in terms of generalizability.

## Data Availability

The original contributions presented in the study are included in the article/supplementary material, further inquiries can be directed to the corresponding author/s.
